# Big Domains Are Novel Ca^2+^-Binding Modules: Evidences from Big Domains of *Leptospira* Immunoglobulin-Like (Lig) Proteins

**DOI:** 10.1371/journal.pone.0014377

**Published:** 2010-12-29

**Authors:** Rajeev Raman, V. Rajanikanth, Raghavan U. M. Palaniappan, Yi-Pin Lin, Hongxuan He, Sean P. McDonough, Yogendra Sharma, Yung-Fu Chang

**Affiliations:** 1 Centre for Cellular and Molecular Biology, Council of Scientific and Industrial Research, Hyderabad, India; 2 Department of Population Medicine and Diagnostic Sciences, College of Veterinary Medicine, Cornell University, Ithaca, New York, United States of America; 3 Institute of Zoology, Chinese Academy of Sciences, Beijing, China; 4 Department of Biomedical Science, College of Veterinary Medicine, Cornell University, Ithaca, New York, United States of America; Instituto Butantan, Brazil

## Abstract

**Background:**

Many bacterial surface exposed proteins mediate the host-pathogen interaction more effectively in the presence of Ca^2+^. Leptospiral immunoglobulin-like (Lig) proteins, LigA and LigB, are surface exposed proteins containing Bacterial immunoglobulin like (Big) domains. The function of proteins which contain Big fold is not known. Based on the possible similarities of immunoglobulin and βγ-crystallin folds, we here explore the important question whether Ca^2+^ binds to a Big domains, which would provide a novel functional role of the proteins containing Big fold.

**Principal Findings:**

We selected six individual Big domains for this study (three from the conserved part of LigA and LigB, denoted as Lig A3, Lig A4, and LigBCon5; two from the variable region of LigA, i.e., 9^th^ (Lig A9) and 10^th^ repeats (Lig A10); and one from the variable region of LigB, i.e., LigBCen2. We have also studied the conserved region covering the three and six repeats (LigBCon1-3 and LigCon). All these proteins bind the calcium-mimic dye Stains-all. All the selected four domains bind Ca^2+^ with dissociation constants of 2–4 µM. Lig A9 and Lig A10 domains fold well with moderate thermal stability, have β-sheet conformation and form homodimers. Fluorescence spectra of Big domains show a specific doublet (at 317 and 330 nm), probably due to Trp interaction with a Phe residue. Equilibrium unfolding of selected Big domains is similar and follows a two-state model, suggesting the similarity in their fold.

**Conclusions:**

We demonstrate that the Lig are Ca^2+^-binding proteins, with Big domains harbouring the binding motif. We conclude that despite differences in sequence, a Big motif binds Ca^2+^. This work thus sets up a strong possibility for classifying the proteins containing Big domains as a novel family of Ca^2+^-binding proteins. Since Big domain is a part of many proteins in bacterial kingdom, we suggest a possible function these proteins *via* Ca^2+^ binding.

## Introduction

Bacterial immunoglobulin-like (Big) folds, also known as Bacterial immunoglobulin-like Domains (BID), are present in many bacterial proteins of 74–90 amino acids in tandem repeats [1]. Proteins containing Big folds range from enzymes to chaperones [2]. Leptospiral immunoglobulin-like (Lig) outer membrane proteins of *Leptospira interrogans* are members of the Big family [3]–[5] that are upregulated during infection of the host and are thought to play a role in the pathogenesis of leptospirosis, a worldwide zoonotic disease [6], [7]. Three related Lig proteins, LigA, LigB and LigC have been identified in *Leptospira*. LigA and LigB have 13 and 12 Big-like domains [8], whereas *ligC* is considered a pseudogene in serovars Grippotyphosa and Copenhageni [3], [9]. However, *ligC* is an intact gene in serovars Kennewicki and Pomona [4], [5], [10].

LigA and LigB (FJ030917 & FJ030916, respectively) proteins have 13 and 12 imperfect tandem repeats of ∼90 amino acids respectively. Both proteins have a highly conserved amino acid sequence at the NH_2_-terminal end but vary at the carboxyl-terminus [3]–[5]. Lig proteins are thought to mediate adhesion of pathogenic leptospires to host cells and thus are possible virulence factors [3]–[5]. The importance of these proteins can be inferred from the fact that they interact with several extracellular matrix proteins, such as fibronectin, elastin, and tropoelastin, which likely aids in host-pathogen interactions [11]–[18]. However, it has been reported that both *ligB* and *ligC* mutants of this organism are still pathogenic and the role of these proteins in the pathogenesis of leptospirosis is still unknown [19]–[21]. Further studies are needed to clarify this issue.

Many surface exposed proteins mediate host-pathogen interactions more effectively in the presence of divalent cations [22]–[25]. Addition of certain inorganic salts to culture medium influences the expression of Lig proteins [26]. Apart from a role in pathogenesis, Ca^2+^ is important in the regulation of diverse bacterial processes such as chemotaxis, cell differentiation and cell division [27], [28]. LigBCen2 consists of the partial 11^th^ and complete 12^th^ immunoglobulin-like repeated domains as well as the first 46 amino acids of the non-repeated region of LigB, has been shown to bind Ca^2+^ [17]. The interaction of LigBCen2 with proteins of the extracellular matrix is enhanced in the presence of Ca^2+^ [17]. It is not known if Big domains of Lig proteins bind Ca^2+^. Answering this question is necessary to understand the Ca^2+^-binding features of Lig proteins, which would eventually help in elucidating their role in the Ca^2+^ dependency of pathophysiology of leptospirosis.

Information about various conformational features of isolated Big domains is rare, although some aspects, such as interactions of Big domains with extracellular matrix proteins, have been described [14]–[17]. The folding pattern of a few immunoglobulin-like proteins has been studied and despite great diversity in structure and function, these domains follow a common protein folding pathway in which conserved residues form a deep hydrophobic folding nucleus [29]. To date, no efforts to study the spectroscopic features of Lig proteins have been reported. The major barrier to such studies is the large size of the protein, which limits the amount of protein that can be prepared from *in vitro* expression systems. However, this limitation can be overcome by studying the individual tandem repeats which will not only increase our understanding of specific features of Lig proteins, but also of Big domains in general.

We have investigated whether individual Big repeats of Lig proteins bind Ca^2+^ and compared the spectral features of selected individual domains. Our data demonstrate that all the selected domains of Lig binds calcium-mimic dye Stains-all, thus demonstrating them as Ca^2+^-binding modules. The domains from the common and variable regions (Lig A3, A4, A9 and A10 along with LigBCen2) bind Ca^2+^ with comparable affinities, suggesting that Big domain containing proteins might belong to a novel family of Ca^2+^-binding proteins. We demonstrate that all three Big domains (Lig A9, Lig A10 and LigBcen2) of Lig studied, have common spectral and conformational features. These Big domains follow common folding pathways and patterns, thus suggesting a similar fold. Understanding Ca^2+^-binding *vis-à-vis* spectral and conformational properties of Lig proteins is of utmost importance in understanding the mechanism of their interaction with host.

## Results

### Domain Selection and Ca^2+^-Binding

**Figure 1:**
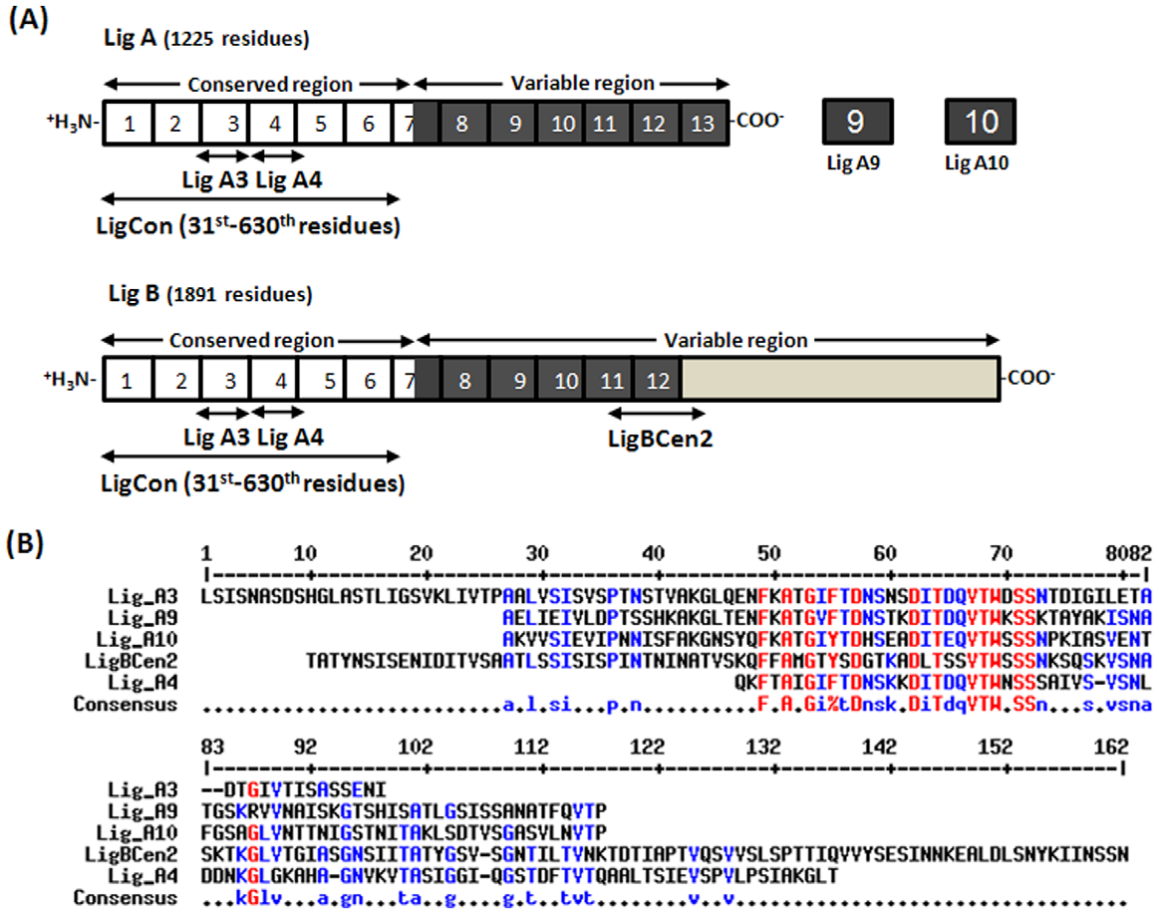
Different domains of Lig and their alignment. (A) Schematic diagram of different domains of LigA and LigB proteins with 13 and 12 Big domain repeats shown with Arabic numerals 1–13. (B) Sequence alignment of the different Lig domains studied using Multalin (URL: http://multalin.toulouse.inra.fr/multalin/). Red and blue colors represent high and low consensus residues (90 and 50%, respectively). In the consensus the symbol, ! is anyone of Ile/Val, and the symbol % represents anyone of Phe/Tyr residue. Lig A3 and Lig A4 are part of the conserved region of LigA and LigB; Lig A9 and Lig A10 have 40% sequence identity whereas LigBCen2 is 37 and 40% identical with the amino acid sequences of Lig A9 and Lig A10. Lig A4 is 46% and 44% identical with Lig A9 and Lig A10 respectively. Arrow indicates the conserved Phe.

LigA and LigB have several tandem repeats of about 90 amino acids which harbor putative Bacterial immunoglobulin fold [5]. To understand if any Big fold of Lig proteins would bind Ca^2+^, we selected several repeats to assay for Ca^2+^-binding (Figure 1A). The selection of domains was made that they cover a bacterial immunoglobulin domain such as repeat 9 as Lig A9, repeat 10 as Lig A10 (91 residues with molecular mass of 9.5 kDa) (Figure 1). We also selected the sequence covering the conserved region of LigA and LigB (LigCon), 1–3 repeat region of LigB (LigBCon1-3), 5^th^ repeat of LigB (LigBCon5), LigBCen2, as well as two domains from conserved region of LigA, repeat 3 as Lig A3, and repeat 4 as Lig A4 as depicted in Figure 1. Lig A9, Lig A10 and LigBCen2 have 37–40% sequence identity between them though LigBCen2 has 17 and 45 extra residues at N- and C-terminal respectively whereas Lig A3 is 68% identical with Lig A9 and 57% with A10.

**Figure 2:**
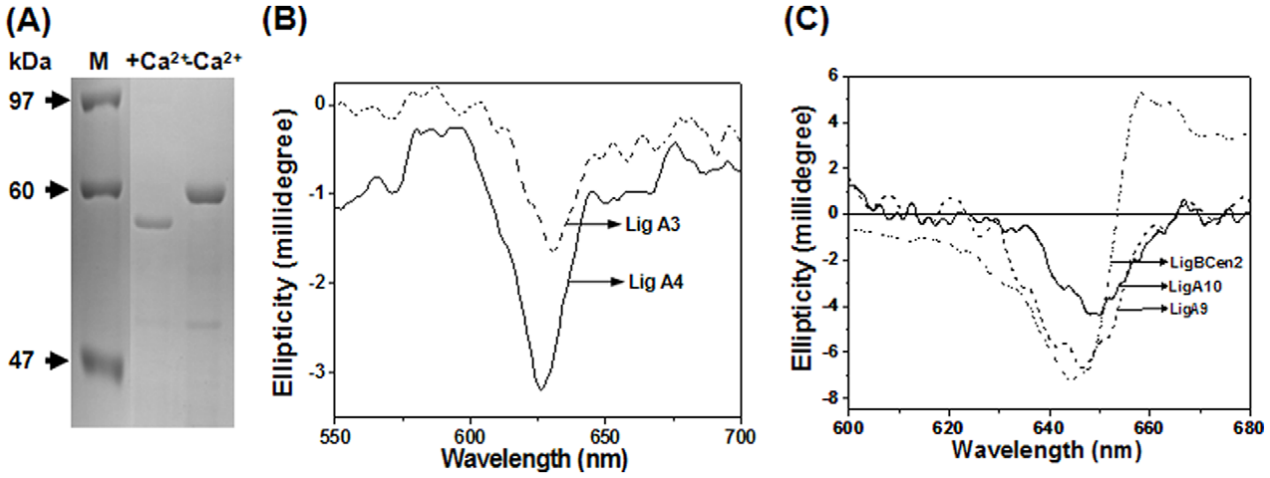
Qualitative Ca^2+^ binding assay. (A) Ca^2+^-dependent gel shift assay on SDS-PAGE. Differential migration was seen when the protein was resolved on a 12% polyacrylamide gel in the presence of 2.5 mM calcium chloride. (B) and (C) represent induced CD spectra of the complexes of Stains-all with LigCon, Lig A3, Lig A4, LigBCon1-3, LigBCon5, LigBCen2, Lig A9 and Lig A10 showing the J band of the dye between 600–650 nm. The J band occurs when the dye is bound to the anionic sites that are present in the globular or compact conformation of proteins. In proteins, where there is no binding, the J band in CD spectrum will not be induced. BSA as a negative control was used in this experiment. Stains-all dye alone has no ellipticity in the visible range.

Many Ca^2+^-binding proteins exhibit mobility differences in the presence of Ca^2+^ when resolved by SDS-PAGE [30]. This assay was used to examine if LigCon (conserved region of LigA and LigB containing 7 tandem repeats) binds Ca^2+^. As seen in figure 2A, LigCon migrated faster in the presence of Ca^2+^ in SDS-PAGE, suggesting the possibility of Ca^2+^-binding. Owing to the low molecular mass of the above selected domains (9.5 kDa), the gel shift assay cannot resolve marginal differential migration in the presence and absence of Ca^2+^. There is no difference in the hydrodynamic volume in the presence and absence of Ca^2+^ as monitored by analytical gel filtration (data not shown). Therefore, Ca^2+^-binding to these individual domains was assayed by Stains-all dye binding method. Stains-all (1-ethyl-2-3-{3-(1-ehtyl-napthol[1,2-d]thiazoline-2-ylidine) -2-methylpropenyl} – naphtho [1,2-d]thiazolium bromide) is a metachromatic cationic carbocyanine dye used as a calcium mimic. The dye which is a racemic mixture does not have any optical activity in visible region. Ca^2+^ -binding proteins induce optical activity in the visible region of the CD spectrum [31], [32]. In this qualitative assay, a Ca^2+^-binding protein may induce any of the five different types of circular dichroism (CD) band in the dye spectrum in the visible range i.e., 400–700 nm [31], [32]. These five band maxima characterized for the dye reviewed earlier are; α band with absorption maxima at 570 nm, the β band at 535 nm, the γ band at 500–510 nm, the J band at 600–650 nm, a mixed band of βα at 550 nm and S band at 470 nm [33]. Similar to other Ca^2+^-binding proteins, all the domains selected from Lig proteins (LigCon, LigBCon1-3, LigBCon5, LigBCen2, Lig A3, Lig A4, Lig A9 and Lig A10) induced the J band of the dye between 600–650 nm, suggesting that selected individual Big domains bind Ca^2+^ (Figure 2B and C).

These results demonstrate that all the domains selected from Lig proteins binds the calcium-mimic dye Stains-all, and thus suggest that they are Ca^2+^-binding modules. We next studied Lig A9 and Lig A10 for detailed Ca^2+^-binding, spectroscopic analysis, and unfolding studies, where Lig A3 and Lig A4 were examined only for Ca^2+^-binding owing to the presence of the GST tag.

### Ca^2+^ Binding and Energetics by Isothermal Titration Calorimetry (ITC)

**Figure 3:**
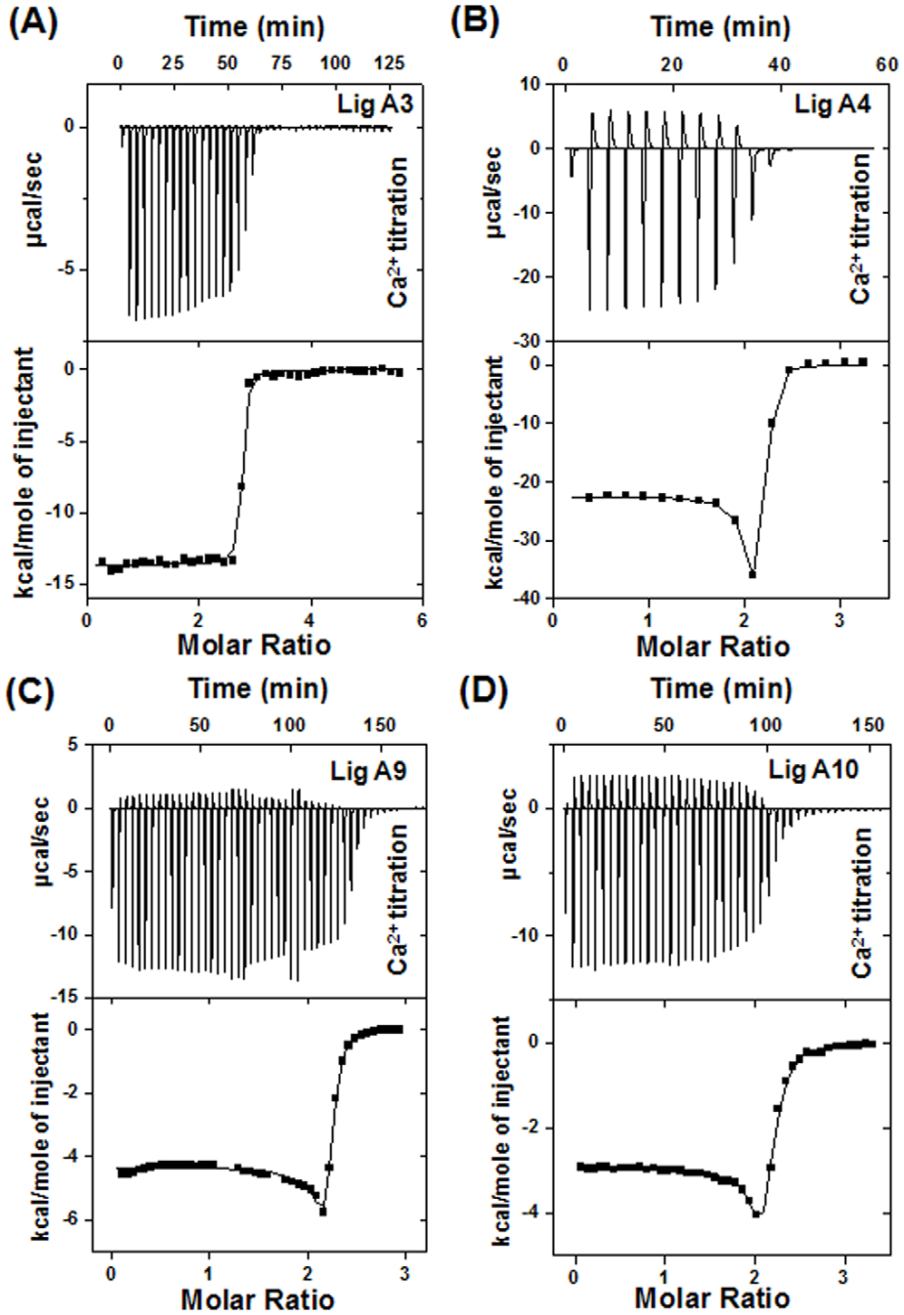
Ca^2+^-binding and stoichiometry of individual Lig domains measured by ITC. A representative titration of Lig A3 (A), Lig A4 (B), Lig A9 (C) and Lig A10 (D) with Ca^2+^ in 50 mM HEPES buffer at 30°C. Sample cell of ITC containing protein was titrated against CaCl_2_. The upper thermogram panel shows the observed heats for each injection of CaCl_2_ at 220 s intervals after baseline correction whereas the lower panel depicts the binding enthalpies *vs.* Ca^2+^/Protein molar ratio. The data (▪) are fitted to best fitted site binding models (A) and (D).

ITC was used to calculate the energetics of Ca^2+^ binding to individual Lig domains at 30°C. Ca^2+^ binding to Lig domains was an exothermic reaction as depicted by thermogram with respective binding isotherm corresponding to a plot of integrated heats as a function of the molar ratio of cations/protein (Figure 3A–D). Ca^2+^ binding to Lig A9 and A10 was driven by favorable enthalpy and unfavorable entropy with over-all dissociation constants (K_d_) of 1.6 µM (K_A_ is macroscopic association constant; K_A1_ = 1.5×10^5^; K_A2_ 2.7×10^6^) and 3.6 µM (K_A1_ = 4.7×10^4^; K_A2_ = 1.6×10^6^) respectively as listed in Table 1. The Ca^2+^-binding isotherm of both Lig A9 and Lig A10 best fitted in two-sets of site model. Binding of Ca^2+^ to Lig A9 and Lig A10 proceeds with a negative enthalpy change (ΔH) which is due to ligand-binding and displacement of water of hydration at binding interface. Lig A3 and A4 also bind Ca^2+^ with dissociation constant (K_d_ values) of 1.4 and 2.2 µM, which is close to that seen for Lig A9 and Lig A10. The binding isotherms indicate release of heat upon Ca^2+^-binding to the protein and the thermodynamic parameters fit results in the values of one set of site for Lig A3 unlike Lig A9 and Lig A10; whereas two sets of sites for Lig A4, probably because of the differences in the protein size.

**Table 1:** Thermodynamic Parameters of Ca^2+^ and Mg^2+^ binding to Lig A3, Lig A4, Lig A9 and Lig A10 at 30°C; calculated by ITC. Ca^2+^-binding was also performed to Mg^2+^-saturated Lig proteins.

Titration	Model	Sites	Macroscopic association constant *K*_A_ (M^−1^)	*K*_d_ a	Δ*H* (kcal/mol)	*T*Δ*S* (kcal/mol)	Δ*G*° (kcal/mol)
Lig A9 *vs* Ca^2+^	Two sets of sites	Set1Set2	K_1_ 1.5×10^5^±7.3×10^5^K_2_ 2.7×10^6^±8.1×10^5^	1.6 µM	−34.7±0.8−4.3±0.03	−27.54.6	−7.2−8.9
Lig A10 *vs* Ca^2+^	Two sets of sites	Set1Set2	K_1_ 4.7×10^4^±5.6×10^3^K_2_ 1.6×10^6^±1.4×10^5^	3.6 µM	−19.8±3.6−2.9±0.01	−13.35.6	−6.5−8.5
Lig A3 *vs* Ca^2+^	One set of site	Set1	7.3×10^5^	1.4 µM	−14.5±0.5	−6.3	−8.1
Lig A4 *vs* Ca^2+^	Two sets of sites	Set1Set2	K_1_ 6.9×10^6^K_2_ 3.1×10^4^	2.2 µM	−22.9±0.31.92±1.6	−13.48.2	−9.5−6.2
Lig A9 *vs* Mg^2+^	One set of site	Set1	3.7×10^5^±4.1×10^4^	2.6 µM	4.2±0.02	11.9	−7.7
Lig A10 *vs* Mg^2+^	One set of site	Set1	1.2×10^5^±4.2×10^3^	8.3 µM	4.4±0.01	11.4	−7.0
Mg^2+^ saturated Lig A9 *vs* Ca^2+^	Two sets of sites	Set1Set2	K_1_ 9.7×10^3^±2.0×10^3^K_2_ 1.0×10^6^±5.7×10^5^	10.0 µM	−10.6±2.7−4.8±0.03	−5.13.5	−5.5−8.3
Mg^2+^ saturated Lig A10 *vs* Ca^2+^	Two sets of sites	Set1Set2	K_1_ 8.7×10^3^±1.8×10^3^K_2_ 2.3×10^5^±7.8×10^4^	22.5 µM	−15.8±7.9−4.6±0.03	−10.32.8	−5.5−7.4

a K_d_ = 1/√ K_1_K_2_, where K_d_ represents the overall binding affinity.

**Figure 4:**
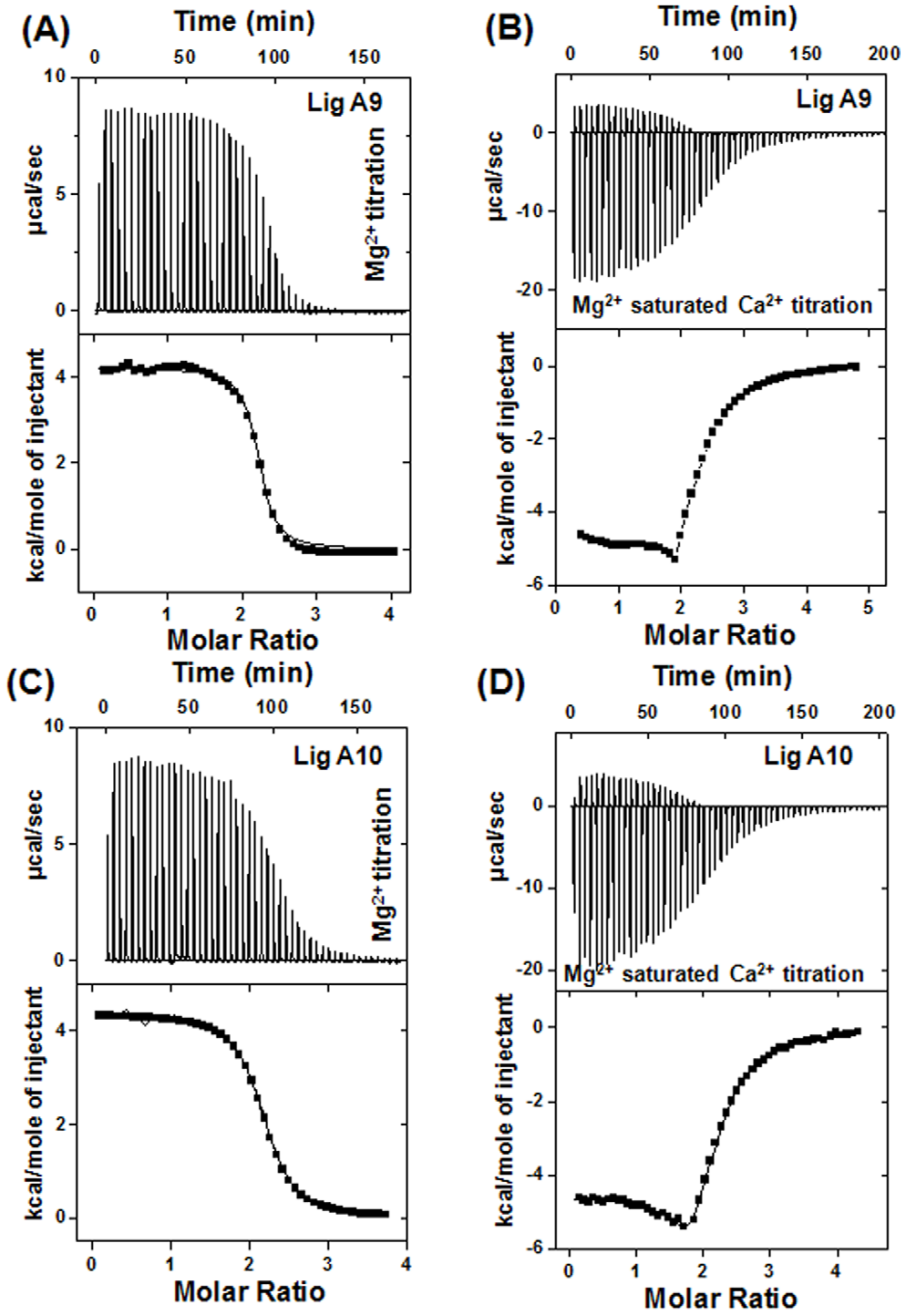
Mg^2+^ binding by Lig A9 and A10 domain. (A) and (C) illustrate MgCl_2_ titration to Lig A9 and Lig A10 proteins. MicroCal LLC ITC software was used for data analysis. In the lower panel of thermogram plot of kcal/mol of heat change per injection of MgCl_2_ as a function of metal: protein is drawn and the least-square fit of the data was considered as the best fit. Data for both proteins fit into a one-set of site model and their affinity for Mg^2+^ is 2.6 and 8.3 µM respectively. Figure (B) and (D) are the ITC profile of Ca^2+^ titration of Mg^2+^-saturated Lig A9 and Lig A10.

Since many Ca^2+^-binding proteins also bind Mg^2+^, we examined Mg^2+^ binding to these domains of Lig proteins. Contrary to Ca^2+^-binding, Mg^2+^-binding to Lig A9 and Lig A10 is an endothermic reaction (Figure 4A and C). Mg^2+^-binding data to apo Lig A9 and Lig A10 fits into one set of site model with at least two Mg^2+^ binding sites with a dissociation constant (K_d_) of 2.6 and 8.3 µM, respectively. In general, Mg^2+^ binding to most of the proteins are entropically driven because of high dehydration enthalpy of divalent cations [30]. The positive enthalpy change (ΔH) of the Mg^2+^ titration to Lig A9 and Lig A10 also helps explain why no large conformational changes were observed during Mg^2+^ titration in CD and fluorescence spectra (Table 1, Figure 4A, C). We further examined Ca^2+^-binding to Mg^2+^-saturated proteins. In these cases, the isotherm exhibits an exothermic reaction. Both Mg^2+^-saturated Lig A9 and Lig A10 bind Ca^2+^ with comparatively less affinity (K_d_ = 10 and 22.5 µM respectively) (Figure 4B, D, Table 1). Mg^2+^ does not bind to Ca^2+^-saturated Lig domains as generally expected for Ca^2+^-binding proteins (data not shown).

### Doublet Emission Spectra of Lig Domains

**Figure 5:**
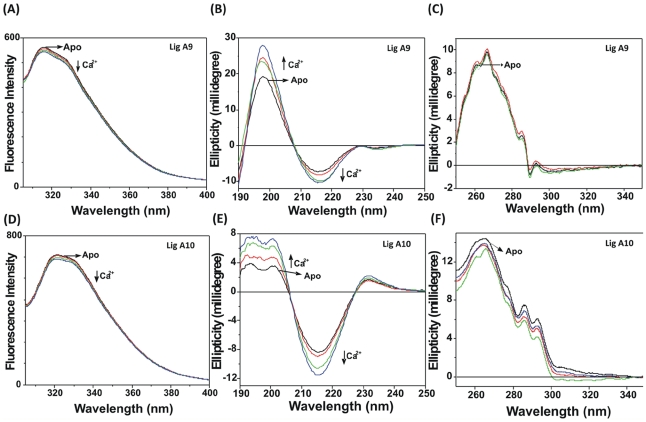
Conformational change of Lig proteins upon Ca^2+^ binding was monitored using Trp emission fluorescence and CD spectroscopy. (A) Steady-state fluorescence spectra, (B) far-UV and (C) near-UV CD spectra of Lig A9 were recorded during titration with Ca^2+^. Decreased fluorescence intensity was accompanied by an increase in ellipticity with the addition of CaCl_2_. (D) Emission spectra of Trp monitored by Fluorescence spectroscopy, (E) far-UV and (F) near-UV CD of Lig A10 was monitored during Ca^2+^ titration. From the figure it is clear that Lig A10 also shows similar change upon Ca^2+^ addition.

Studying the fluorescence spectra of Lig A9 and Lig A10 was of interest since both the domains have single tryptophan. Excitation of Lig A9 at 295 nm produced an atypical emission spectrum with a doublet (two peaks) at 316 and 327 nm (Figure 5A). A similar fluorescence spectrum with two unresolved but clear peaks was obtained in the case of Lig A10, but with both peaks red shifted to 320 and 330 nm (Figure 5D). We obtained similar doublet spectra in case of other domains as well as LigCon (data are not shown as the emission spectra of all the domains are almost similar to that shown in Figure 5). The origin of such emission spectra with two peaks has been explained earlier due to a hydrophobic interaction between a Trp and Phe [35]. Such wave length maxima (λ_em, max_) are obtained if a Trp of a protein is surrounded by largely non-polar environment. When both Lig domains were titrated with increasing concentrations of Ca^2+^, a nominal decrease in fluorescence intensity was observed suggesting that no significant change in conformation occurs upon Ca^2+^-binding (Figure 5A, D). Lig A9 and A10 do not undergo any change in fluorescence spectra with MgCl_2_ (data not shown). We also investigated surface hydrophobicity of these domains of Lig proteins using 1-anilino-8-naphthalene sulfonic acid (ANS) as a fluorescence probe. Neither Lig A9 nor Lig A10 binds ANS, suggesting that these proteins are likely to be highly hydrophilic (these data are not shown).

### Ca^2+^-Binding Induces an Increase in the Magnitude of Ellipticity

To determine the effect of Ca^2+^ on the secondary and tertiary structure of Lig A9 and A10, we examined far- and near-UV CD spectroscopy in the presence and absence of Ca^2+^. The far-UV CD spectrum of Lig A9 with a broad negative peak at 217 nm and a strong positive peak at 198 nm is indicative of a typical β-sheet conformation (Figure 5B). Lig A10 also displays a similar pattern in the far-UV CD region (Figure 5E) suggesting that both Lig domains are largely in β-sheet conformation. These proteins were titrated with 40, 80 and 100 µM of CaCl_2_ and far-UV CD spectra were recorded. There was about 10–15% increase in the magnitude of positive and negative ellipticity at 198 and 217 nm in Lig A9 upon Ca^2+^ addition (Figure 5B). A similar increase in the magnitude of the negative peak was observed in Lig A10, suggesting the same conformational changes in both proteins upon Ca^2+^ binding (Figure 5E). Mg^2+^ did not induce any change in the far-UV CD spectra of either Lig protein (data not shown).

Lig A9 and A10 have three Phe, one Trp and one and two Tyr residues respectively. Near-UV CD spectra of both domains has positive signals for Trp at 294 and 288 nm, for Tyr at 278 nm and a strong peak for Phe at 268 nm and a hump at 262 nm suggesting that despite separating from a parent protein, both domains fold well (Figure 5C, F). A strong ellipticity peak for Phe at 268 nm suggests that Phe residues are either immobilized or interacting with neighboring residues, in agreement with the fluorescence data which showed a doublet peak at around 320 and 330 nm. Upon addition of Ca^2+^, only marginal changes in ellipticity at 288 and 192 nm were seen (more prominent in Lig A10), typically associated with a Trp residue where as the 278 nm peak is because of Tyr.

### Big Domain Homodimerization

As seen by CD and fluorescence data, both domains have a well defined structure. We next examined by gel filtration if these domains would self associate to form dimers or high order oligomers. Under both conditions (with and without Ca^2+^), Lig A9 and A10 elute from a gel filtration column at a molecular mass of approximately 18 kDa, while LigBCen2 elutes at around 33 kDa (Figure S1A, B) confirming that Lig proteins dimerize in a Ca^2+^-independent manner. Since there is no Cys residue, it is obvious that these proteins do not dimerize *via* any disulphide bridges. The homodimerization is not protein concentration dependent, suggesting their inherent nature of domain self-association.

### Thermal Stability Measurement by CD and Differential Scanning Calorimetry (DSC)

**Figure 6:**
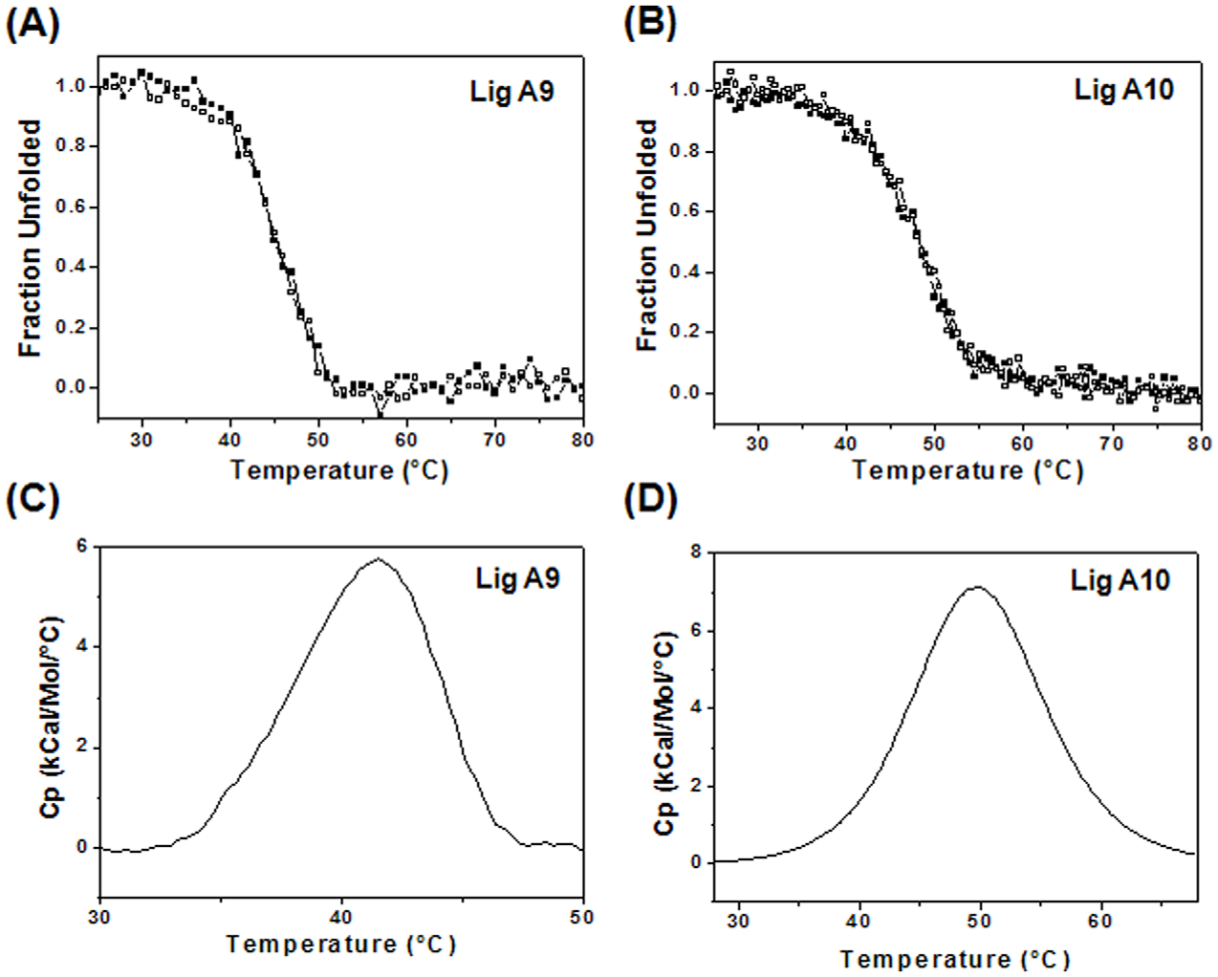
Stability of Lig proteins. (A) Thermal unfolding of Lig A9 measured at pH 7.0 with 50 mM KCl and monitored by CD spectroscopy at 225 nm with (▪) and without Ca^2+^ (□). T_m_ was increased at 2°C increments in the presence of Ca^2+^. (B) Temperature-dependence of CD spectra of Lig A10 in the far-UV regions in the presence (▪) and absence (□) of CaCl_2_. The temperature was increased from 25°C to 80°C at 1°C/min and ellipticity *vs.* temperature was plotted. No change in T_m_ was observed. Molar heat capacity *vs.* temperature was plotted in the presence of 2 mM CaCl_2_ for (C) Lig A9, and (D) Lig A10 by DSC.

CD and DSC were used to examine the thermal stability of Lig proteins. The change in ellipticity was measured at 225 and 229 nm for Lig A9 and A10 and the mid-point of thermal unfolding transition was calculated. Both domains are moderately stable with a midpoint of unfolding (T_m_) for Lig A9 at 45.4°C for the apo form, with a marginal increase for the holo form (T_m_ 47.5°C) (Figure 6A). T_m_ obtained for Lig A10 was 47.9°C (in both apo and holo forms) (Figure 6B). Native conformation was recovered after cooling of the sample suggesting that no irreversible reactions had taken place.

T_m_ is the temperature at which the excess heat capacity, C_p_, is at its maximum. We compared the thermal unfolding and stability by DSC. T_m_ of Lig A9 was 40.8±0.66 (in apo) and 41.1±0.7°C (in Ca^2+^-bound form), whereas T_m_ for Lig A10 was 49.8±0.02°C in both cases (Figure 6C, D). DSC data along with CD suggest that although Ca^2+^ binds to the proteins, it does not influence their thermal stability.

### Equilibrium Unfolding Monitored by Intrinsic Fluorescence

**Figure 7:**
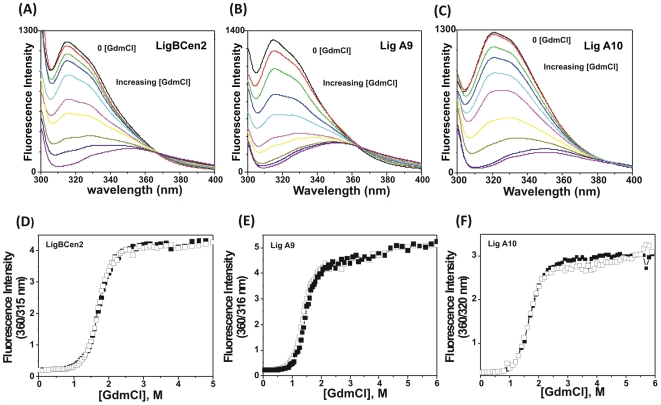
Chemical unfolding monitored by fluorescence. Trp emission spectra in increasing concentrations of GdmCl in 25 mM HEPES buffer containing 50 mM KCl were recorded at 295 nm excitation. (A) LigBcen2, (B) Lig A9 and (C) Lig A10 in the presence of 0, 0.5, 0.65, 0.8, 0.9, 1.05, 1.15, 1.35 and 1.55 M of GdmCl. Concentration of GdmCl *vs*. the ratio of fluorescence intensities at 360 nm and λ_max_ (of native protein) as shown in graph (D) for LigBCen2, (E) Lig A9 and (F) Lig A10. Equilibrium unfolding transitions are plotted in the presence (▪) and absence of Ca^2+^ (□) using 315 and 320 nm fluorescence intensities.

To examine stability, the unfolding of Lig A9, Lig A10 and LigBCen2 was monitored by Trp fluorescence and CD (near- and far-UV) with increasing concentrations of guanidine hydrochloride (GdmCl). When GdmCl was added to Lig proteins, the fluorescence intensity of the lone Trp decreased significantly (∼70%) surprisingly without any red shift in the wavelength maxima. Up to ∼1.3 M of GdmCl concentration resulted in no observed change in the wavelength maxima and the characteristic plateau between 315 and 330 nm was still intact (Figure 7A, B and C). Beyond 1.3 M of GdmCl, the characteristic plateau started disappearing with a concomitant red shift in the spectra. With further addition of GdmCl (>2 M GdmCl), the emission peak shifted towards 350 nm with a further decrease (up to >80%) in intensity, suggesting that the proteins were unfolded at this concentration of GdmCl. Further addition of GdmCl did not bring any change in the emission spectra (Figure 7A, B and C). To examine if this pattern is denaturant dependent, we also followed unfolding by urea. A similar decrease in the emission intensity without any red shift was also observed up to 1.5 M of urea (beyond this concentration, red shift of the emission towards 350 nm was observed) suggesting that the pattern of unfolding is denaturant independent (Figure S2).

**Figure 8:**
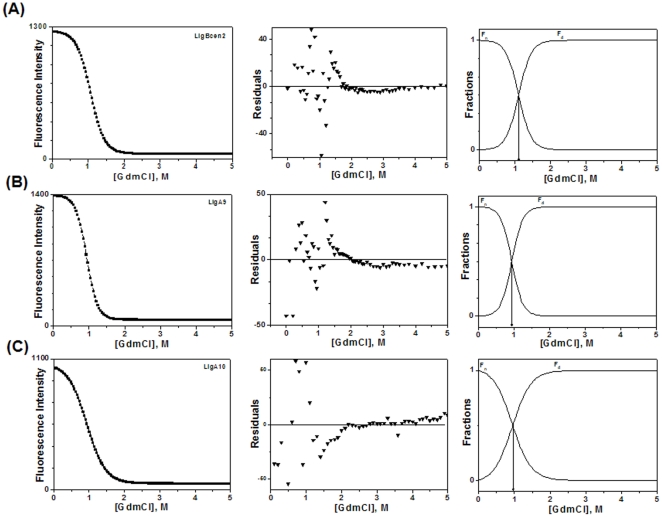
Equilibrium unfolding transition of Lig proteins by guanidinium chloride (GdmCl). Unfolding was measured by fluorescence intensity at 315 nm for (A) LigBCen2 and (B) Lig A9; and 320 nm for (C) Lig A10 with excitation wavelength of 295 nm for all three domains. Residual fitting for each protein are in the middle column that validates the data fitting. The last column shows the distribution of native and unfolded state of Lig proteins as a function of [GdmCl]. The fractions of native (fn) and denatured (fd) protein for all three Lig proteins were deduced from the unfolding transitions.

We monitored fluorescence intensity both at 320 or 330 nm and the unfolding transition curves were drawn and it is evident from Figure S3 that regardless of monitoring fluorescence intensity at 320 or 330 nm as shown for Lig A10 are super-imposable. A similar trend was also noticed for other Lig domains (Lig A9 and LigBCen2). The ratio between 360/320 nm fluorescence intensity versus GdmCl concentration graph was plotted and it demonstrates a shift in emission maxima upon unfolding (Figure 7D, E and F). The ratio method is less susceptible to small changes in protein concentration, but due to the nonlinearity of the curve, we used fluorescence intensity at 315 nm for Lig A9 and LigBCen2; and 320 nm for Lig A10 for the data fitting. In both conditions (apo and holo), data were best fitted to a two-state transition model without any intermediate and steep sigmoidal curves, indicating a high degree of co-operative unfolding (Figure 8). Lig proteins are highly hydrophilic and no change in surface hydrophobicity was observed by ANS binding up to 2 M of GdmCl concentrations (Data not shown). Free energy change (ΔG°) of the unfolding process of LigBCen2 was 3.67±5.70×10^−6^ kcal/mol which was close to the values obtained for Lig A9 as shown in Table 3 with a D_1/2_ (midpoint concentration of GdmCl) of 1.10 M and 0.93 M, respectively. Lig A10 has a lower ΔG° value (2.13±0.20 kcal/mol with D_1/2_ of 0.96 M). Upon Ca^2+^ addition, no significant change in Gibbs free energy was observed in any Lig protein, suggesting that Ca^2+^ does not act as an extrinsic stabilizer.

### Unfolding of Big Domains Monitored by CD

**Figure 9:**
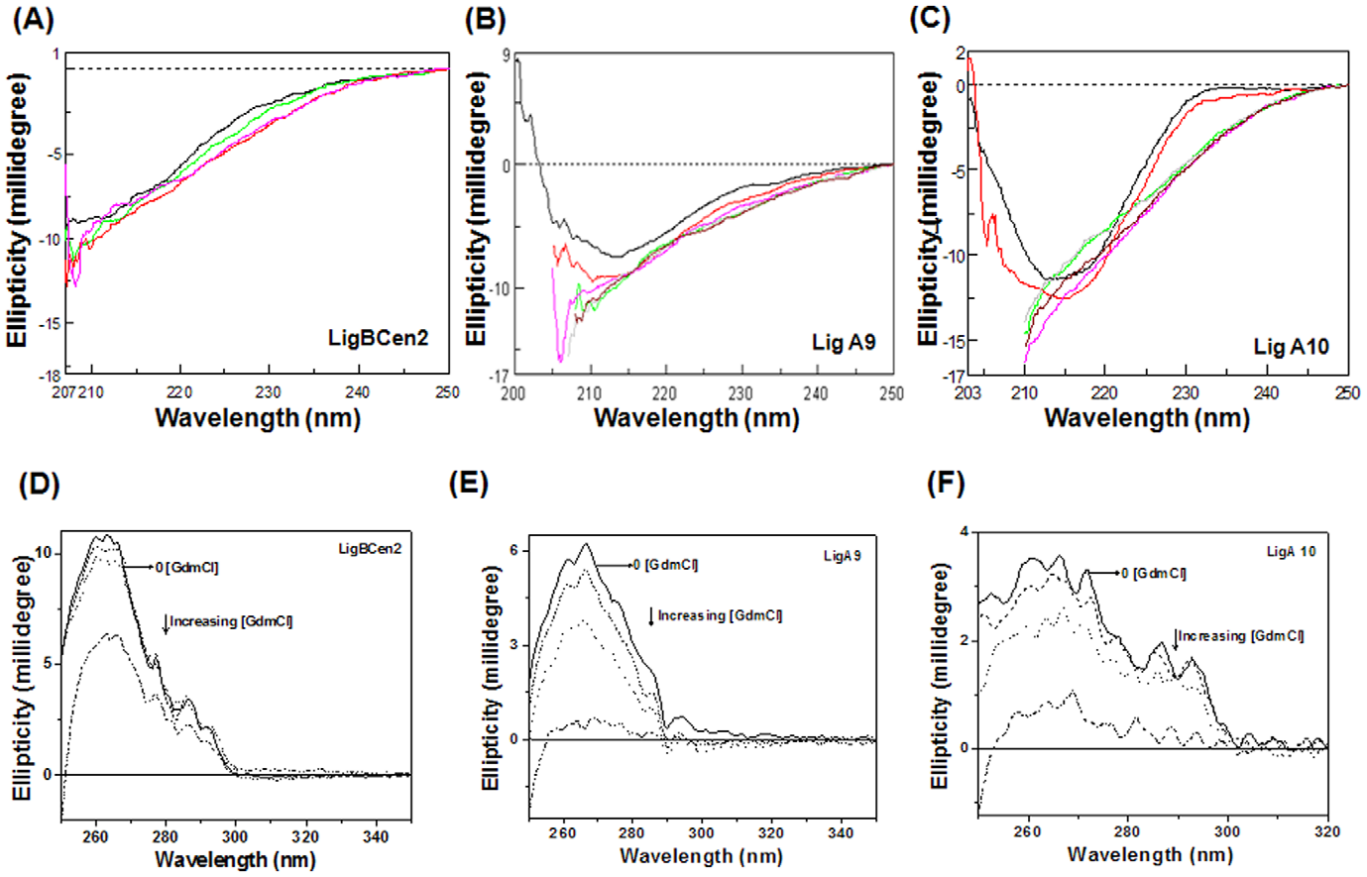
Change in CD spectra of Lig proteins by GdmCl. Far-UV CD spectra of (A) LigBCen2 (B) Lig A9 and (C) Lig A10 in the presence of 0, 0.65, 1.15 and 1.65 M of GdmCl in 25 mM HEPES buffer containing 50 mM KCl. Near-UV CD spectra of (D) LigBCen2, (E) Lig A9 and (F) Lig A10 were recorded in the presence of 0, 0.5, 1.0 and 2.0 M of GdmCl concentrations.

Since there was a drastic decrease in fluorescence emission intensity, we monitored the change in secondary and tertiary structure of Lig proteins by near- and far-UV CD spectra. For GdmCl concentrations up to 1.5 M, far-UV CD spectra of Lig proteins, as depicted in figure 9A, B and C, are similar to the native protein spectra, indicating that no global secondary structural change occurred. Beyond 1.5 M GdmCl, secondary structure was almost completely lost. High concentrations of GdmCl interfered with the collection of CD data in the far-UV region. Near-UV CD spectra were also monitored for change in tertiary structure by increasing the concentration of GdmCl. With increasing GdmCl concentrations, a slow loss in tertiary structure was recorded, and even 0.5 M was enough for inducing a change in tertiary structure, and beyond 2 M of GdmCl complete loss in tertiary structure was found (Figure 9D, E and F).

## Discussion

No prior data are available on the spectral properties of an individual bacterial immunoglobulin-like domain. The results of this study provide insight into the conformational features of individual Big domains of these Lig proteins which may be important host-pathogen interactions. Our results demonstrate a significant level of conformational similarity between various Big domains of Lig proteins, despite only moderate sequence similarities. These domains fold well as individual units, as seen from their near-UV CD spectra, and have largely β-sheet conformation. All individual Big domains of Lig proteins form homo-dimers, probably *via* domain pairing. Homodimerization of individual domains (such as in case of βγ-crystallin domain) have been linked for the evolution of multi-domain proteins *via* gene fusion [34]. Fluorescence spectra of both individual domains are unusual as they show a doublet, not generally shown by a typical protein Trp (Figure 5). Another Lig domain LigBCon4 also exhibited similar doublet spectra as shown earlier [15]. The origin of a doublet peak could be possibly due to a hydrophobic interaction between a Trp and a Phe in the excited state as demonstrated in the case of RNase [35]. Both Lig A9 and Lig A10 have a single Trp at position 43 and it is likely that upon excitation; this Trp might form a complex with Phe23. This Phe is conserved between three domains and there are 21 residues in between Trp and Phe (Figure 1B).

Reshetnyak and Burstein [36] have classified Trp in a protein into five different classes on the basis of emission maxima, quantum yield and spectral bandwidth at the half-maximal amplitude, Δλ. Trp with the most blue-shifted emission maxima (λm  = 316 nm) belongs to class S, which forms exciplexes with water or neighboring protein groups in a 1∶1 stoichiometry in the excited state. Prominent peaks of Trp fluorescence for all three Lig domains (Lig A9, Lig A10 and LigBcen2) are found at 316, 320 and 317 nm with another peak/hump at 330 nm. Δλ measurement of Lig proteins is not possible because of highly blue-shifted spectra and doublet. Only a few proteins with such a doublet spectra, one being a δ-crystallin, are documented [37]. Therefore, we group the Trp of these proteins along with δ-crystallin from chick and hen lens (Trp with doublet peak as R class) under a separate R class, different from class S and class I.

The most interesting property exhibited by the Big domains of Lig proteins is Ca^2+^-binding. These proteins also bind Mg^2+^, surprisingly with higher affinity than the proteins of the EF-hand family [38]. The binding of ligand does not introduce extensive change in ellipticity or fluorescence. Near-UV CD shows strong evidence of tertiary structure and no significant change was noted upon Ca^2+^ binding. High affinity towards Ca^2+^ and moderate change in conformation suggest that binding of Ca^2+^ is largely structural; though earlier studies show Ca^2+^ enhances the interaction of Lig with extracellular matrix proteins [17]. Though only a few properties of Lig proteins are known, the ability of these Big domain containing proteins to bind Ca^2+^ was recently reported [17]. Only a single domain, LigBcen2, was seen to bind Ca^2+^ raising the question for further analysis if other domains also bind Ca^2+^. We further demonstrate that any Big repeat of either Lig proteins (LigA or LigB) should bind Ca^2+^. The variation seen in the binding affinity might be due to the difference in the size of the proteins. We extrapolate our data to suggest that Big domains from other proteins should have the similar fold topologies as shown by these Big domains of Lig proteins. The importance of this work lies in the fact that these proteins do not have any known Ca^2+^-binding motif (such as Greek key βγ-crystallin fold, C2 domain or EF-hand) and thus the actual motif needs to be identified.

Like Big domains, numerous other unclassified Ca^2+^-binding consensus regions have been predicted earlier in other bacterial proteins, such as Cah, an autotransporter protein from enterohaemorrhagic *E. coli* [39]. A cell surface protein of *Staphylococcus aureus* binds calcium at its repeat segments, which has a sequence signature similar to an EF-hand loop [40]. A calcium-dependant cell surface bacterial protein was found to be involved in the attachment of rhizobia to peanut roots, suggesting a Ca^2+^-dependent role of surface proteins [24]. Ca^2+^-binding proteins in bacteria with EF-hand and other motifs such as present in toxins have been explored [18]. Recently, LipL32, an outer surface protein from *Leptospira* spp., was also found to bind to Ca^2+^ [41], [42]. This work provides a more general view of Ca^2+^-binding to a large group of proteins, i.e., Big domains.

The immunoglobulin-like fold is somewhat similar to a Greek key βγ-crystallin motif with no specifically conserved functions [43]. The Greek key βγ-crystallin fold forms a motif for Ca^2+^-binding and a number of non-lens proteins possessing this fold have been shown to bind Ca^2+^ [44], [45]. Another well-known Ca^2+^-binding motif, the C2 domain of protein kinase C, is also a β-sheet motif [46]. The Ca^2+^-binding motif of Big is also a β-sheet conformation (probably distantly related with a Greek key type fold), adding to the list of Greek key Ca^2+^-binding motifs though there might not be any other sort of similarities. More studies are required to understand the molecular nature of Ca^2+^-binding to these proteins though interaction of Lig proteins with extracellular matrix proteins is enhanced in the presence of Ca^2+^, thus implicating the Ca^2+^-protein interaction in pathophysiology and virulence [17].

Sequence similarities between these tandem repeats, which fold as a domain, are seen from their spectral properties. The interesting feature of unfolding is the concomitant decrease in fluorescence intensity (without any shift in emission maxima) in the presence of GdmCl or urea. Though there is a sharp decrease in emission intensity, it is due to the unfolding of the proteins (and not a simple quenching) as confirmed by the loss of tertiary structure seen by near-UV CD. Red-shift in the spectra is seen when GdmCl concentration is over 1.6 M. All three domains studied followed a similar pattern of unfolding with more or less similar stability suggesting common structural elements that are the possible signature of the fold. We raise the question if other domains of Lig proteins should also exhibit similar patterns of unfolding. This unfolding profile could be used to compare diverse Big domains from other proteins.

In conclusion, the spectral as well as unfolding patterns of these Big domains suggest several interesting features, including a unique fluorescence emission and a specific unfolding pattern. Ca^2+^-binding suggests a new role for these domains, not only in the case of Lig proteins, but also in case of other proteins in the same superfamily. This work suggests the strong possibility that Lig proteins, along with other surface exposed proteins and proteins containing Big domains, form a new class of Ca^2+^-binding proteins.

## Materials and Methods

### Gene Constructs, Bacterial Strain and Culture Conditions of Lig proteins

Various regions of Lig proteins (FJ030917 and FJ030916 for LigA and LigB, respectively) used in this study are illustrated in Figure 1A and were cloned from the genomic DNA of *L. interrogans* serovar Pomona. Briefly, the PCR products of LigCon (first 599 residues of common region of LigA and B), Lig A3 (residues 281–378 of common region) and Lig A4 (common region residues 379–466) were cloned into SmaI and XhoI restriction sites of pGex4T-2 vector (Amersham Biosciences) as GST fusion.

The 9^th^ and 10^th^ tandem repeats of 91 amino acids (nucleotides 2551–2823 and 2824–3096) of LigA (Lig A9 and Lig A10) were amplified from *L. interrogans* genomic DNA *via* PCR using primers listed in Table 2. The resulting DNA was ligated into pET22b vector (Novagen Inc. WI, USA) under T7 promoter and transformed into *E. coli* BL21 (DE3). The transformed *E. coli* cells were grown to mid-log phase in 2X YT media and heterologous protein expression was induced by 1 mM isopropylthio-β-D-galactoside (IPTG). The cultures were harvested for 7 h after induction. Vector-construct, over-expression and purification of LigBCen2, LigBCon1-3 and LigBCon5 have been described earlier [5], [11], [15].

**Table 2:** DNA sequence of the primers used for PCR amplification of Big domains of Lig proteins. Underline sequence represents the restriction endonuclease site used for cloning.

Primer	Primers' sequence
LigCon Forward	TCCCCCGGGGCTGGCAAAAGA
LigCon Reverse	CCCTCGAGAATATCCGTATTAGA
Lig A3 Forward	AACCCGGGGGAATATTAGAAA
Lig A3 Reverse	AACTCGAGTTAGTTGAGTGTGG
Lig A4 Forward	TCCCCGGGGGGAATGTTAAAGTCA
Lig A4 Reverse	GTCTCGAGTTAAGCGTGAGCTT
Lig A9 Forward	CATATGGCGGAACTTATTGAG
Lig A9 Reverse	CTCGAGTTACGGAGTAACTTGGAA
Lig A10 Forward	CATATGGCTAAAGTAGTTTCGATCG
Lig A10 Reverse	CTCGAGTTATGGATGACATTCAA

**Table 3:** Thermodynamic parameters for GdmCl-induced unfolding of Lig proteins.

Proteins	ΔG° (kcal/mol)	m (kcal/mol/M)	D_1/2_
Lig A9	3.65±6.49×10^−6^	3.886±6.45×10^−6^	0.93 M
Lig A10	2.13±0.20	2.259±0.17	0.96 M
LigBCen2	3.67±5.70×10^−6^	3.344±4.88×10^−6^	1.10 M

The unit for ΔG° is kcal/mol and for m is kcal/mol/M. D_1/2_ is the midpoint concentration of GdmCl.

### Purification of various Big domains of Lig protein

LigCon protein was overexpressed in LB medium (with 50 mg/ml ampicillin) after induction with 1 mM IPTG. Protein was purified using glutathione-Sepharose 4B affinity column (Amersham Biosciences) as a GST fusion protein. Only in case of LigCon, GST fusion was removed by thrombin in thrombin cleavage buffer (20 mM Tris-HCl, pH 8.4, 150 mM NaCl, 2.5 mM CaCl_2_) for 16 h incubation at room temperature. Thrombin was removed using benzamidine-Sepharose (Amersham Biosciences) and cleaved GST was removed by FPLC using Superose 12 (Amersham Bioscience) column.

Similarly, Lig A3 and Lig A4 were also purified as GST tagged proteins, whereas Lig A9 and Lig A10 were overexpressed without any tag. The over-expressed Lig A9 and Lig A10 were induced using 1 mM IPTG purified from the soluble fraction. The supernatant was treated with ammonium sulphate by a two-step salting out procedure at 4°C. The ammonium sulphate precipitation obtained at 25% concentration was discarded. Lig proteins were precipitated at 55% final concentration of ammonium sulphate. The precipitate thus obtained was collected and dissolved in 25 mM HEPES buffer (pH 7.0). Final purification was performed on a Sephadex G-75 gel filtration column in 25 mM HEPES buffer, pH 7.0, containing 50 mM KCl. Buffers were decalcified by passing through a Chelex-100 resin column (Bio-Rad) whereas all proteins were decalcified by treating with 2 mM ethylenediaminetetraacetic acid (EDTA) for one hour followed by buffer exchange on a 3 kDa cut-off Amicon stirred cell as confirmed by atomic absorption spectroscopy. Protein concentration was estimated using the theoretical extinction coefficient, calculated by ProtParam tool from ExPASy (URL: http://expasy.org/tools/protparam.html; last access June 2010).

### Ca^2+^ Dependent Mobility Shift of LigCon by SDS-PAGE

Generally, there is a differential migration of a Ca^2+^-binding protein in the presence of Ca^2+^ which can be resolved in the SDS-PAGE [30]. Briefly, 10 µg of purified LigCon was incubated for 5 min with 2.5 mM CaCl_2_ or 5 mM EDTA, boiled for a few minutes and subjected to 12% SDS-PAGE. The protein bands were visualized by staining with Coomassie Brilliant Blue.

### Calcium-Binding by ITC

VP-ITC calorimeter (MicroCal) was used for all ligand binding studies and data were analyzed by MicroCal LLC ITC software (MicroCal). Decalcified protein samples prepared in decalcified 25 mM HEPES buffer (pH 7.0) containing 50 mM KCl were thoroughly degassed before use. For the Ca^2+^-binding experiments, protein samples (50, 50, 450 and 500 µM of Lig A3, Lig A4, Lig A9 and Lig A10) were titrated against 5, 5, 10 and 10 mM CaCl_2_ respectively, all in the same buffer (25 mM HEPES, pH 7.0, 50 mM KCl) at 30°C. After reaching saturation with CaCl_2_, titration with 5 mM MgCl_2_ was performed. Typically, 60 injections of 5 µl each were made at 220 sec intervals. Heat change was recorded as differential power by the instrument and determined by integration of the obtained peak. Titration of ligand to buffer was performed and subtracted from the sample to correct for the heat of dilution. Data of corrected heat change were fitted using the nonlinear least square method to obtain parameters like binding enthalpy (ΔH), dissociation constant (K_d_) and stoichiometry.

### Conformational Studies by Fluorescence

Fluorescence emission spectra of Lig A9 and Lig A10 proteins were recorded on a Hitachi F-4500 spectrofluorometer (Hitachi, San Jose, CA). All spectra were recorded in the correct spectrum mode with both excitation and emission slits set at 5 nm. Intrinsic fluorescence spectra of Lig proteins were recorded by exciting the solution at 295 nm and emission spectra were recorded from 300 to 400 nm in 25 mM HEPES, pH 7.0 containing 50 mM KCl. For Ca^2+^ and Mg^2+^ titration, 0.1, 0.2, 0.5 and 1 mM standard CaCl_2_ or MgCl_2_ solutions were mixed and incubated for 3 minutes before recording a spectrum.

### Circular Dichroism (CD) Spectroscopy

CD spectra were recorded on a Jasco J-815 spectropolarimeter (JASCO Corporation, Tokyo, Japan) at room temperature. The path lengths used were 0.02 and 0.5 cm for far- and near-UV CD measurement respectively. All spectra were recorded in 25 mM HEPES buffer containing 50 mM KCl with appropriate protein concentrations. The baseline spectra of buffer without protein were subtracted from respective protein spectra. All the CD spectra are represented as the ellipticity in millidegrees.

For the Stains-all dye binding experiment, Lig proteins (LigCon, LigBCon1-3, LigBCon5, Lig A3, Lig A4, Lig A9, Lig A10 and LigBCen2) were mixed with 100 µM dye solution prepared in 2 mM MOPS buffer, pH 7.2, containing 30% ethylene glycol and CD spectra were recorded from 400 to 700 nm after a 10 min incubation in the dark as described earlier [31], [32]. Stains-all dye being a racemic mixture does not show any band in CD. Bovine serum albumin was used as a negative control.

Thermal unfolding of Lig A9 and Lig A10 was monitored by measuring the ellipticity by CD at a fixed wavelength (225 or 229 nm) as a function of temperature from 25 to 80°C with 1°C/min increments. The reversibility of protein unfolding was examined by scanning the same sample and recording the CD spectra during cooling by a built-in temperature control unit.

### Differential Scanning Calorimetry

Calorimetric measurements were performed using a VP-DSC and MicroCal LLC DSC software was used for data acquisition and analysis. The samples and references were degassed immediately before use. About 15 µM of Lig A9 or Lig A10 in 25 mM HEPES buffer with and without 2 mM CaCl_2_ were used. Temperature scans were performed from 25 to 85°C at a scan rate of 1°C/min and data were corrected for buffer base line prior to concentration normalization.

### Size Exclusion Chromatography

For homodimerization, size exclusion chromatography of Lig proteins (Lig A9, Lig A10 and LigBCen2) were performed on a pre-packed Superose 12 column (10/300 GL column, GE Healthcare) in 25 mM HEPES buffer pH 7.0, 50 mM KCl (in the case of LigBCen2, 50 mM Tris buffer, pH 7.0, containing 50 mM KCl was used) with and without 3 mM CaCl_2_. For comparison of molecular mass of Lig proteins, the gel filtration column was calibrated with standard protein molecular mass markers.

### Equilibrium Unfolding Measurement of Big-like domains by Fluorescence and Data Analysis

Chaotropic agent guanidine hydrochloride (GdmCl) was used for unfolding studies. The GdmCl concentration was calculated from the refractive index of the solution using the equation [GdmCl]  = 57.147 (ΔN) + 38.68(ΔN)^2^ - 91.60(ΔN)^3^, where ΔN is the difference in refractive index between the denaturant solution and buffer on the refractive indices of aqueous solutions of GdmCl [47]. For proper equilibrium unfolding transitions, a minimum of 70 points were taken [48]. Increasing concentrations of GdmCl (8 M stock) were added to Lig A9, Lig A10 and LigBCen2 (70, 8 and 20 µM, respectively) to achieve required (ranging from 0 to 6 M) concentrations of denaturant in the presence of 2 mM CaCl_2_ or EDTA. The mixtures were incubated overnight at room temperature to achieve equilibrium. The GdmCl induced unfolding transitions were followed at 295 nm excitation. Change (decrease) in fluorescence intensity and wavelength shift from the native state were measured and data fitting was performed using various models. The best fitted model was the two-state model (N↔U) where N and U are the native and unfolded state of the protein. Equation 1 was deduced for the two-state model by nonlinear regression analysis. For thermodynamic parameters such as free energy change (ΔG°) from native to unfolded state, and ‘m’, which represents the free energy dependence on denaturant concentration associated with unfolding, were determined using Graph Pad Prism software version 4 (GraphPad Inc., San Diego, CA, USA).

### Equation 1: two-state equation



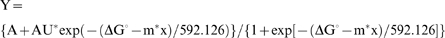



## Supporting Information

Figure S1 Lig proteins form homo-dimers. (A) Gel filtration FPLC of LigBCen2 and LigA10. The protein and molecular mass standard were applied to a Superose 12 column in a 50 mM Tris buffer, pH 7, containing 50 mM KCl and elution volumes were measured. (B) Elution profile of LigA9 and standard protein markers from a Superose 12 column equilibrated with a 25 mM HEPES buffer (pH 7.0), containing 50 mM KCl. All proteins elute at a dimerization elution volume.
(1.54 MB DOC)

Figure S2 Chemical unfolding monitored by fluorescence. Emission spectra of (A) LigBcen2 (B) LigA9 and (C) LigA10 in presence of 0, 0.1, 0.25, 0.35, 0.5, 0.75, 1.0, 1.25, 1.5, 1.75, 2.0, 2.5, 3.0 M of urea in 25 mM HEPES buffer containing 50 mM KCl. Similar pattern of decrease in fluorescence intensity as shown in figure 7 was followed in presence of urea, suggest that unfolding is denaturant independent. Though it was obvious protein unfold in more urea concentration compare to GdmCl.
(9.93 MB TIF)

Figure S3 GdmCl unfolding of Lig A10. There are two emission picks of LigA10, at 320 (▪) and at 330 (□) nm. There is no difference in unfolding pattern of LigA10 whatever peak is chosen. Same pattern was also observed for LigA9 and LigBCen2.
(1.38 MB TIF)
