# Supplementary figures and images for: Big Domains Are Novel Ca2+-Binding Modules: Evidences from Big Domains of Leptospira Immunoglobulin-Like (Lig) Proteins
Source: PLoS One. 2010 Dec 29;5(12):e14377. doi: 10.1371/journal.pone.0014377 (PMC3012076; doi:10.1371/journal.pone.0014377)

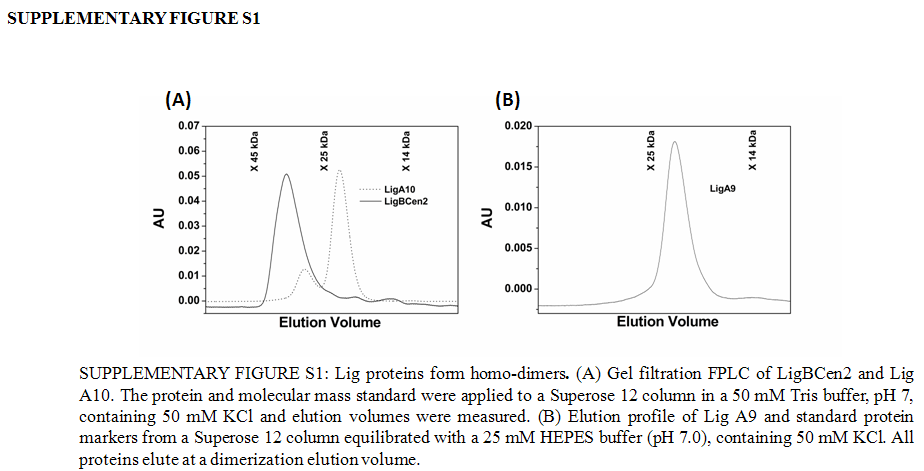

Supplement: Figure S1 — Lig proteins form homo-dimers. (A) Gel filtration FPLC of LigBCen2 and LigA10. The protein and molecular mass standard were applied to a Superose 12 column in a 50 mM Tris buffer, pH 7, containing 50 mM KCl and elution volumes were measured. (B) Elution profile of LigA9 and standard protein markers from a Superose 12 column equilibrated with a 25 mM HEPES buffer (pH 7.0), containing 50 mM KCl. All proteins elute at a dimerization elution volume. (1.54 MB DOC) [file pone.0014377.s001.tif]

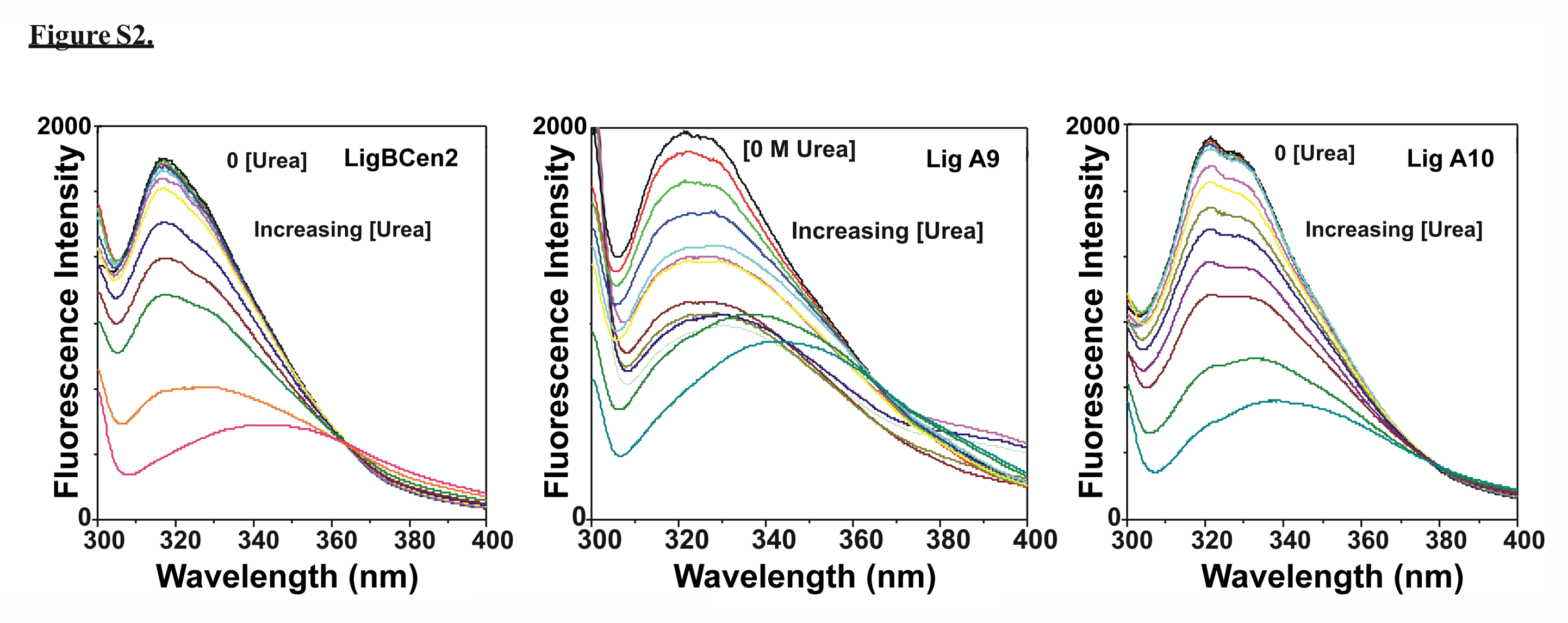

Supplement: Figure S2 — Chemical unfolding monitored by fluorescence. Emission spectra of (A) LigBcen2 (B) LigA9 and (C) LigA10 in presence of 0, 0.1, 0.25, 0.35, 0.5, 0.75, 1.0, 1.25, 1.5, 1.75, 2.0, 2.5, 3.0 M of urea in 25 mM HEPES buffer containing 50 mM KCl. Similar pattern of decrease in fluorescence intensity as shown in figure 7 was followed in presence of urea, suggest that unfolding is denaturant independent. Though it was obvious protein unfold in more urea concentration compare to GdmCl. (9.93 MB TIF) [file pone.0014377.s002.tif]

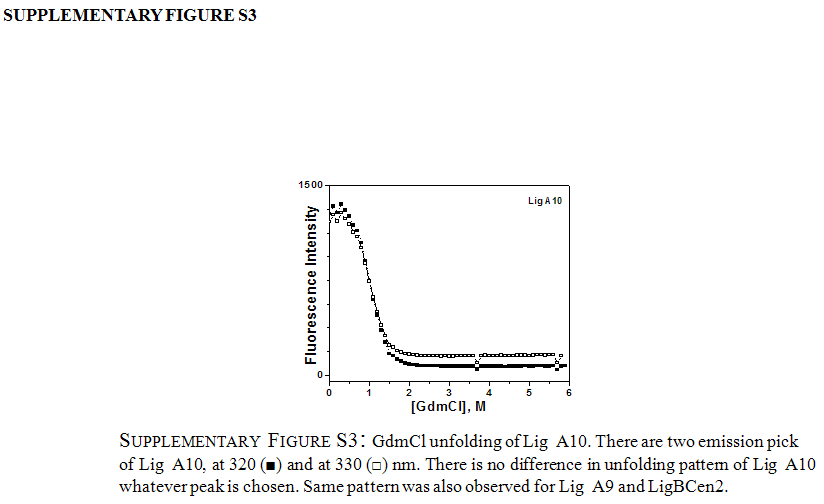

Supplement: Figure S3 — GdmCl unfolding of Lig A10. There are two emission picks of LigA10, at 320 (▪) and at 330 (□) nm. There is no difference in unfolding pattern of LigA10 whatever peak is chosen. Same pattern was also observed for LigA9 and LigBCen2. (1.38 MB TIF) [file pone.0014377.s003.tif]
